# Discovery of Nuclear DNA-like RNA (dRNA, hnRNA) and Ribonucleoproteins Particles Containing hnRNA

**Published:** 2016

**Authors:** G.P. Georgiev

**Affiliations:** Institute of Gene Biology, Russian Academy of Sciences, Vavilova Str., 34/5, Moscow, 119334, Russia

## Abstract

On August 9–11, 2014, Cold Spring Harbor (USA) hosted a special symposium dedicated to the discovery of messenger or informational RNA and the main events in the subsequent studies of its synthesis, regulation of synthesis, maturation, and transport. The existence of mRNA in bacteria was first suggested in 1961 by Jacob and Monod, based on genetic studies [1]. The same year, Brenner* et al*. confirmed the hypothesis [2]. Our laboratory played a key role in the discovery of messenger RNA in eukaryotes, as well as in the discovery of the nuclear ribonucleoproteins that contain it and in the elucidation of their structural organization. Therefore, I was invited to represent Russia at the Symposium and deliver a speech on these topics. However, my visa had only been issued after the end of the Symposium, and, therefore, the presentation was delivered by my former colleague G.N. Yenikolopov, who works at Cold Spring Harbor Laboratory. The transcript of the lecture is presented below.

## DISCOVERY OF NUCLEAR DRNA

The research discussed in this paper was initiated in my group at I.B. Zbarsky Laboratory at the A.N. Severtsov Institute of Animal Morphology of the Soviet Academy of Sciences. However, the bulk of the research was conducted in my lab at the Institute of Molecular Biology of the Soviet Academy of Sciences, to which I was invited by its director, V.A. Engelhardt, whose name the Institute now bears.

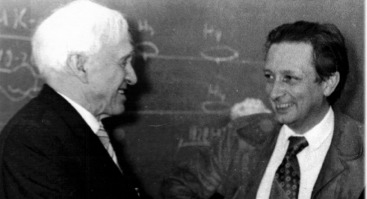



Director of the Institute of Molecular Biology, RAS, V.A. Engelhardt with the author

**Fig. 1:**
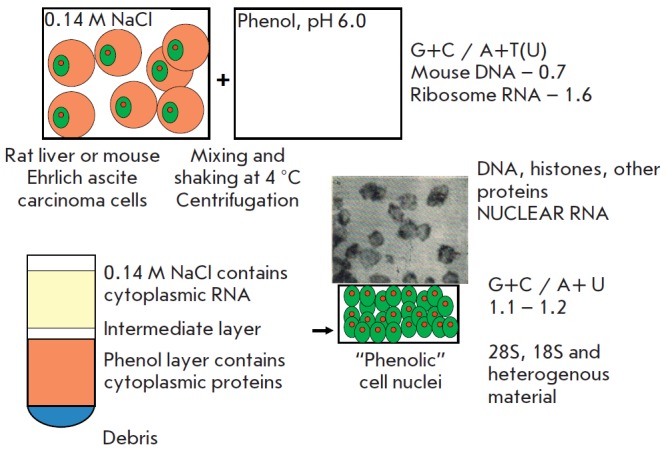
Isolation and properties of “phenolic” cell nuclei. (left panel) Scheme of cell nuclei isolation by phenol treatment. A photograph of the “phenolic” nuclei of Ehrlich ascites carcinoma cells is presented. (right-hand panel) Composition of the obtained nuclei and properties of their RNA: intermediate nucleotide composition between those of DNA and rRNA; ultracentrifugation data.

My main collaborator in the discovery of nuclear dRNA was V.L. Mantieva, who went on to earn a PhD in biology. We were interested in the nature of nuclear RNA [3] and used a newly developed phenol method to isolate RNA from cells [4]. A suspension of mouse Ehrlich ascites carcinoma cells was shaken in 0.14 M NaCl and phenol at pH 6.0 and 4°C, followed by centrifugation. Surprisingly, in addition to the expected aqueous and phenol phases, the centrifugation produced an intermediate layer that contained cell nuclei that retained their shape [5]. These nuclei contained chromatin and nucleoli, which stored DNA, nuclear RNA, and most of the nuclear proteins (*Fig. 1*). Since phenol inhibits enzyme activity, we believed that “phenolic” nuclei can be a good source of nuclear RNA. Later, it was shown that nuclear RNA can indeed be extracted from “phenolic” nuclei by this procedure if it is performed at 65°C [3]. The isolated nuclear RNA contained components with sedimentation coefficients of 28S and 18S, typical for ribosomal RNA, and heterogeneous material. The nucleotide composition of the nuclear RNA was intermediate between mouse DNA (G+C/A+T = 0.72) and ribosomal RNA (G+C/A+U = 1.65) (*Fig. 1*). It seemed that nuclear RNA contained ribosomal RNA and a new type of RNA whose nucleotide composition was similar to that of DNA: i.e., informational RNA. The first experiments on the fractionation of nuclear RNA, conducted in 1961, confirmed this hypothesis [3].

The best separation was achieved by thermal phenol fractionation, developed in 1962, that included treatment of “phenolic” nuclei with a 0.14 M NaCl–phenol mixture at pH 6.0 and stepwise increase in temperature [6]. At 40°C, the aqueous phase contained pure RNA whose nucleotide composition corresponded to ribosomal RNA (rRNA) and which contained a precursor of ribosomal RNA. At 55 to 65°C, the aqueous phase contained pure RNA with a nucleotide composition similar to that of DNA (G+C/A+U = 0.7–0.74). Notably, ^32^P-labeled RNA experiments revealed that the nucleotide compositions of the total RNA of the isolated fraction and that of the nascent RNA present in it were identical [7, 8] (*Table*). The discovered and purified DNA-like RNA was named dRNA. Three years later, in 1965, American authors described this type of RNA and called it heterogeneous nuclear RNA (hnRNA) [9-12].

**Table:** Isolation of nuclear DNA-like RNA by phenolic thermal fractionation

RNA (DNA) Fraction	G	C	A	U(T)	G+C / A+U(T)
Mouse DNA	21	21	29	29	0.72
Cytoplasmic, 4°C	32	30	20	18	1.63
Nuclear, 4–40°C	32	29	20	19	1.50
Nuclear, 55–65°C	23	20	28	29	0.76
Nascent nuclear, 55–65°C	21	20	29	30	0.71

**Fig. 2:**
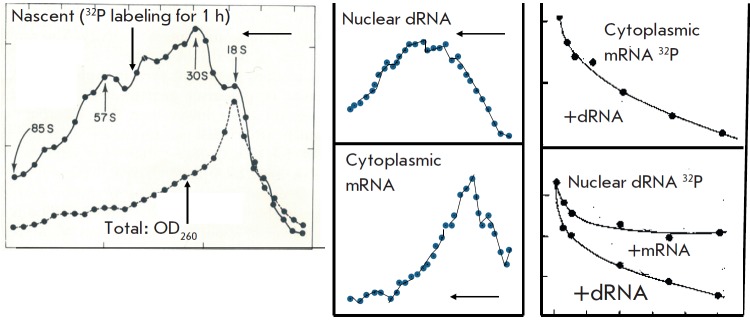
Characteristics of nuclear dRNA. (left-hand panel) Ultracentrifugation of nuclear dRNA labeled for 1 h with ^32^P in a sucrose density gradient. Significantly higher molecular weight of the labeled dRNA than that of total dRNA as determined by optical density. Here and later, thin arrows indicate the direction of ultracentrifugation. (central panel) Comparison of the molecular weights of nuclear dRNA and cytoplasmic mRNA labelled in identical conditions (1 h). The former has a much higher molecular weight. (right-hand panel) Hybridization of labeled cytoplasmic mRNA and nuclear dRNA with DNA and competition with unlabeled nuclear dRNA and cytoplasmic mRNA

Next, we described the properties of nuclear dRNA. Its molecular mass was highly heterogeneous and reached very high values. The nascent nuclear dRNA had a significantly higher molecular mass than the total nuclear dRNA, which implied its cleavage in the cell nucleus (processing) [7, 8] (*Fig. 2*).

We also determined the size of dRNA in the cytoplasm, presumably, mature mRNA. We developed a method of partial blocking of RNA synthesis by actinomycin D, which in small doses selectively inhibits rRNA synthesis without affecting dRNA synthesis. The molecular weight of the nascent nuclear dRNA significantly exceeded that of cytoplasmic dRNA [7, 8] (*Fig. 2*).

Finally, we conducted DNA hybridization-competition experiments with nuclear dRNA and cytoplasmic mRNA. The addition of nuclear dRNA completely inhibited mRNA hybridization with DNA, whereas an excess of mRNA only partially inhibited hybridization of nuclear dRNA with DNA (*Fig. 2*).

We hypothesized that nuclear dRNA is a high-molecular mass precursor of cytoplasmic mRNA, or pre-mRNA, which is partially destroyed during dRNA processing and mRNA maturation that occurs in the cell nucleus, from which mRNA is exported to the cytoplasm. Definite proof of this hypothesis required several years of research by a number of laboratories, but the initial confirmation of the existence of messenger RNA in eukaryote cells had been presented in the above mentioned papers [6-8].

## DISCOVERY OF RIBONUCLEOPROTEIN (RNP) PARTICLES: DRNP (HNRNP)

Our next goal was to elucidate the organization of nuclear dRNA in the cell nucleus. My main collaborator in this work was O.P. Samarina, who later became doctor of biological sciences, professor, and a Lenin Prize winner.

A mild procedure was used to study hnRNA-containing structures. Rat liver nuclei were treated with 0.14 M NaCl, 1 mM MgCl_2_, and 10 mM Tris buffer at pH 7.0. A portion of RNA was extracted, and its nucleotide composition was found to be intermediate between rRNA and dRNA. Three subsequent extractions with the same solution at pH 7.8–8.0 solubilized a significantly higher proportion of RNA that had the same nucleotide composition as pure dRNA. A DNA-like composition was typical of both the total and the nascent RNA in the extract [13].

**Fig. 3:**
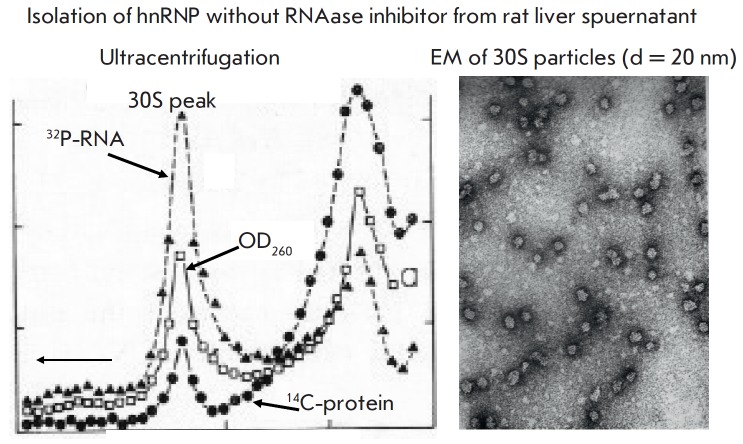
Properties of the nuclear hnRNP particles obtained at the first stage of the research. (left-hand panel) Sucrose gradient ultracentrifugation of nuclear extracts containing hnRNA. RNA was labeled with ^32^P-orthophosphate and a protein with a mixture of ^14^C-amino acids. (right-hand panel) Electron microscopy of 30S particles from the sucrose gradient

**Fig. 4:**
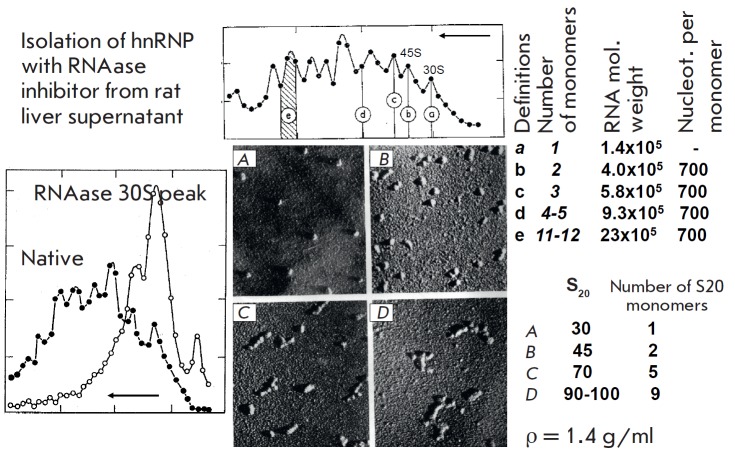
Properties of nuclear hnRNP polyparticles. (left panel) Distribution of hnRNP particles isolated with a RNAase inhibitor at ultracentrifugation. Conversion of polyparticles into 30S monomers with mild RNAase treatment. (top panel) Polyparticles, molecular weights of RNA extracted from particles with different numbers of monomers, and the number of nucleotide monomers per particle. (bottom right panel) EM of the particles from different areas of the gradient, sedimentation coefficients, and number of monomers. The buoyant density is the same for all particles (1.4)

After ultracentrifugation, most of the hnRNA was detected in a homogeneous 30S peak, which contained particles ca. 20 nm in diameter. The molecular weight of RNA isolated from the 30S peak was low (*Fig. 3*). It was contrary to the data on the very high molecular mass of hnRNK isolated by phenol fractionation. To resolve this contradiction, we performed extraction in the presence of a RNAase inhibitor from rat liver supernatant. This extraction produced a completely different pattern of ultracentrifugation: a series of peaks ranging from a small 30S peak all the way up to material with sedimentation coefficients of 200S and above (*Fig. 4*). Obviously, this pattern was much closer to the native one [14].

Notably, both the 30S peak and the peaks with higher molecular masses had the same buoyant density in CsCl (after formaldehyde fixation): ca. 1.4 g/ml, which corresponds to a RNA/protein ratio of about 1:4–1:5.

Next, we characterized the larger particles. Mild ribonuclease A treatment quantitatively transformed them into 30S particles with 20-nm diameter, which were, therefore, monomers of the larger polyparticles. Electron microscopy demonstrated that 30S particles were monomers, 45S particles were dimers, 70S particles were pentamers, and that the 90–100S peak contained polyparticles built up of 9 monomers. The measurement of RNA obtained from various peaks demonstrated that in all cases the monomer was a RNA fragment ca. 700 nucleotides in size. This is consistent with the fact that the buoyant density of all hnRNP peaks was identical (*Fig. 4*).

Thus, hnRNP are chains built up of similar RNP particles connected by RNA bridges, which are the most sensitive to ribonuclease treatment [15].

**Fig. 5:**
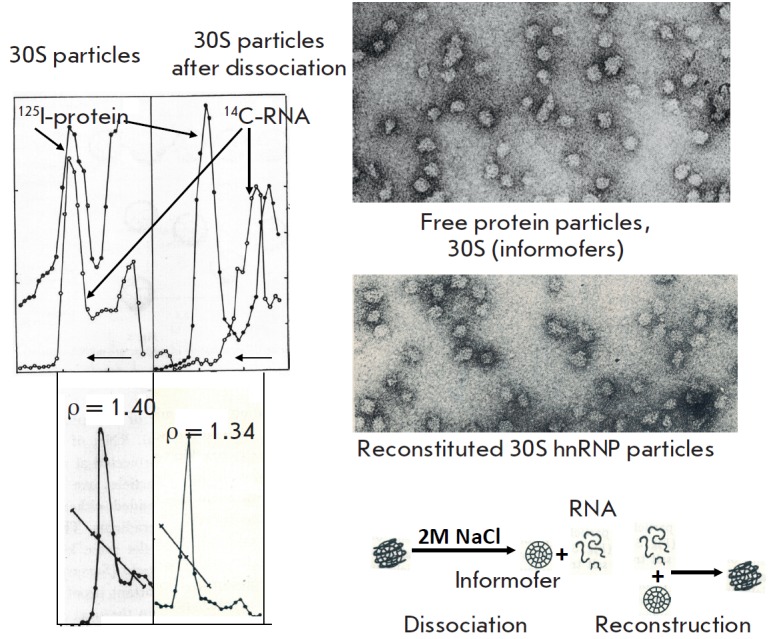
Structure of hnRNP particles. (right-hand panel) RNA- and protein-labeled 30S particles (the latter labelled with ^125^I) before and after treatment with 2M NaCl. In contrast to the initial particles, those treated with 2M NaCl lost all total RNA, although their sedimentation coefficient and EM dimensions remained unchanged. Buoyant density decreased from 1.4 to 1.34 g/ml. (lefthand panel) EM of dissociated and reconstructed 30S particles. (bottom panel) The scheme of dissociation and reconstruction of hnRNP.

To better understand the structure of hnRNP particles, we elucidated the structure of the 30S monomer. Intense ribonuclease treatment completely destroyed the 30S particle RNA, which suggests that it is localized on the particle’s surface. The 30S proteins were labeled with ^125^I, and the particles were treated with 2M NaCl to cause dissociation of RNA and the protein. After ultracentrifugation, all of the hnRNA remained in the upper fractions, whereas the protein was detected in the same 30S peak, despite the removal of RNA. The buoyant density of 30S particles dropped to 1.34 g/cm^3^ [1-6] (*Fig. 5*).

When protein particles were mixed with hnRNA and 2 M NaCl was removed by dialysis, hnRNP particles were reconstructed and were indistinguishable from the initial ones in a variety of tests. The initial 30S particles, protein particles, and reconstructed hnRNP particles look the same in electron microscopy (*Fig. 5*). In the presence of hnRNP of about 1.4 kDa in size, dimeric hnRNPs are formed during the reconstruction [15]. The protein 30S particles were called ‘informofers’ (messenger RNA carriers), but this term failed to gain traction in the literature.

Informofers are protein complexes containing ca. 20 protein molecules with a molecular weight of about 40 kDa, belonging, according to other authors’ data, to six different types [16]. It was concluded that nuclear hnRNP particles are long hnRNA chains regularly wrapped on the surface of a series of similar or identical protein globular particles. This structure significantly reduces the size of long hnRNA, while leaving it available for interaction with more specific factors involved in RNA processing and export.

Interestingly, a similar principle of organization was later discovered for chromatin nucleosomes [17].

## FURTHER MRNA STUDIES AT THE INSTITUTE OF GENE BIOLOGY, RAS

This author moved on to other topics related to the organization of genome (discovery and characterization of mobile genetic elements in animal cells) and chromatin. However, regulation of hnRNA synthesis and mRNA export has been actively studied at the Institute of Gene Biology of the Russian Academy of Sciences that was organized 15 years ago and for which it is the main focus of research. Another important area of research at the Institute is new approaches to cancer therapy. This author is now engaged is this research. Some key studies related to mRNA are summarized below.

**Fig. 6:**
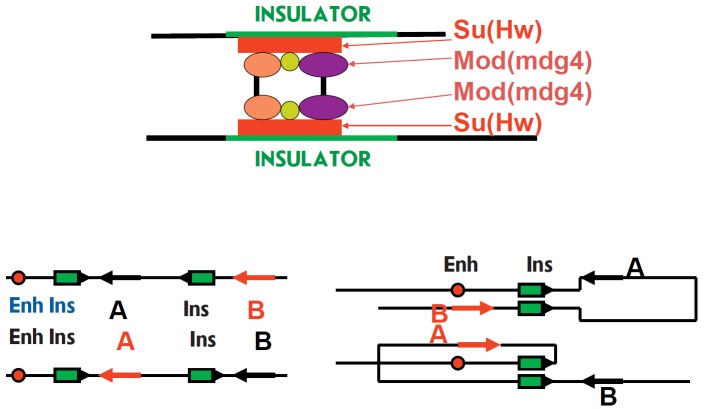
Insulator interactions. Due to the presence of a series of proteins, multiple contacts are formed in the insulator protein complex and they define strong binding between insulators and the polarity of their interaction. Only similarly aligned insulators can bind to each other, which defined the configuration of the loop and activation of a gene

First of all, new properties belonging to insulators, important* cis*-elements in transcription regulation, have been discovered. Their role has been found to be highly dependent on their ability to bind tightly to each other [18, 19]. This property depends on the dimerization of a number of proteins that make up insulator complexes: e.g., the Mod(mdg4) protein discovered at the Institute [20] (*Fig. 6*).

In contrast to enhancers, insulators are polar; they only interact with each other if they have the same orientation. This determines conformation of a loop formed as a result of insulators interaction and may define which genes will be activated [21, 22] (*Fig. 6*).

**Fig. 7:**
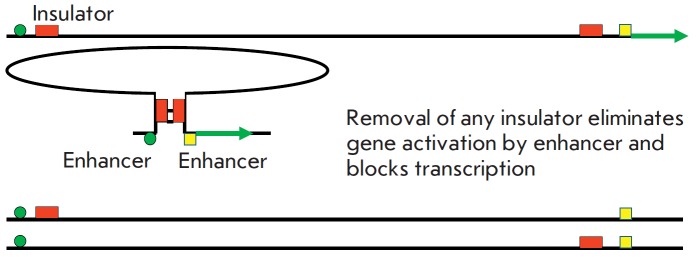
The super-long-distance interaction in the genome. They are defined by the interaction of insulators and can lead to activation of a promoter by an enhancer. Removal of any one or two insulators prevents this interaction

Super-long-distance interactions have been discovered in the genome [23, 24]. They can reach dozens of millions of base pairs and can occur even between non-homologous chromosomes. They depend on the interaction between insulators and can lead to activation of a promoter by an enhancer. Removal of one of the insulators results in a complete loss of super-long-distance interaction, which manifests itself as inactivation of the associated transcription (*Fig. 7*).

**Fig. 8:**
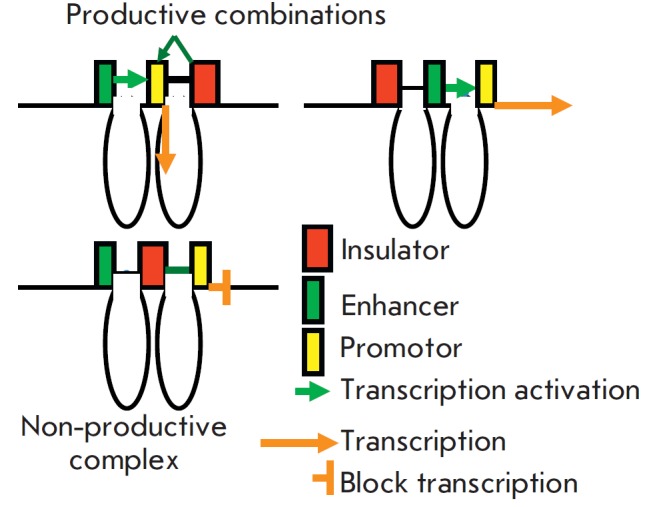
Interaction of insulators with other elements. Insulators interact with promoters and activate them and with enhancers (more selectively). If an insulator is located between them and there is no other insulators nearby, it may interact with both, forming a non-productive complex

Finally, it was discovered that insulators can interact with promoters (with low selectivity), activating them, and with enhancers (more selectively). Therefore, an insulator located between an enhancer and a promoter may interact with both, forming a non-productive complex. This may explain the well-known uncoupling effect of an insulator [25, 26] (*Fig. 8*).

Two new proteins, E(y)2/ENY2 and SAYP, have been discovered. They play an important role in the control of hnRNA transcription and subsequent stages of mRNA formation and export [27, 28].

**Fig. 9:**
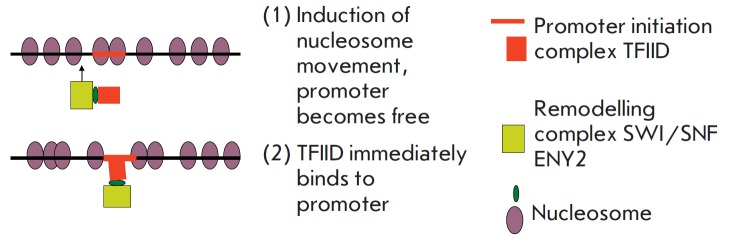
Scheme of the supercomplex with the SAYP protein. The formation of the supercomplex dramatically increases the efficiency of TFIID binding with a promoter and transcriptional activity

SAYP binds the protein initiating complex TFIID and chromatin remodeling complex SWI/SNF into a single supercomplex. Knockdown SAYP blocks the recruitment of TFIID and SWI/SNF at the promoter and represses the transcription of many genes. It can be assumed that the fusion of the complexes allows TFIID to immediately bind to the promoter as soon as the SWI/SNF-activated movement of nucleosomes along the DNA release it from the nucleosomes [29] (*Fig. 9*).

**Fig. 10:**
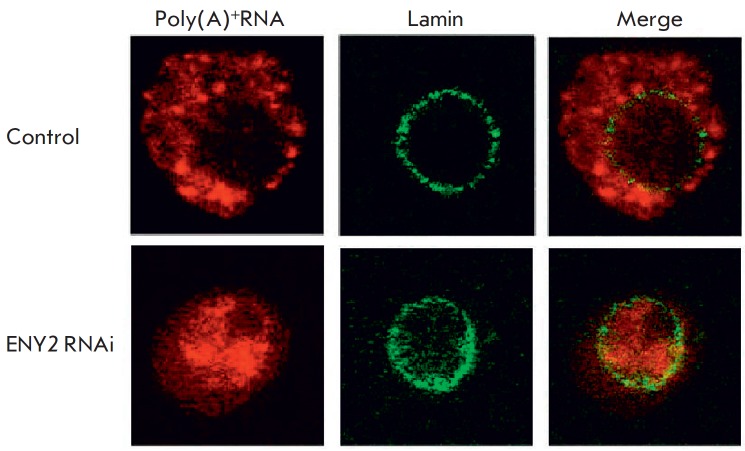
ENY2 protein knockdown blocks mRNA export from the nucleus to the cytoplasm. Inhibition of protein synthesis is achieved via RNA interference. As a result, almost all of the poly(A)+RNA remains in the nucleus

E(y)2/ENY2 was found to be multifunctional. It is part of the DUB module of the SAGA complex and participates in the activation of transcription initiation [30]. ENY2 is also part of the THO protein complex involved in hnRNA elongation, binding to hnRNP and export of some mRNAs. ENY2 is also an important component of the Drosophila AMEX protein complex, binding hnRNP and playing a key role in the export of many mRNAs. Knock down of ENY2 by RNA interference leads to complete blockage of mRNA transport from the nucleus to the cytoplasm. All mRNA accumulate in the nucleus [31] (*Fig. 10*). Finally, ENY2 is part of some insulator complexes, performing the barrier function of insulator [32].

This is only part of the Institute’s work in the field of regulation of mRNA synthesis and export.

Therefore, early work on the identification of messenger RNA in eukaryotes successfully continues.
